# Super-additive multi-dimensional self-balancing deficit: implication for flight mishaps and falling

**DOI:** 10.1007/s00221-026-07395-7

**Published:** 2026-09-16

**Authors:** Vivekanand P. Vimal, Paul DiZio, Avijit Bakshi, Alberto Pierobon, Henry P. Williams, James R. Lackner

**Affiliations:** 1 Ashton Graybiel Spatial Orientation Laboratory, Brandeis University, Waltham, MA USA; 2 Department of Psychology, Brandeis University, Waltham, MA USA; 3 Volen Center for Complex Systems, Brandeis University, Waltham, MA USA; 4 Acceleration and Sensory Sciences Department, Naval Medical Research Unit – Dayton, Wright Patterson AFB, OH USA

**Keywords:** Dynamic balance, Manual guidance, Pendulum, Multisensory, Orientation, Flight mishap, Falling, Decision error

## Abstract

We report super-additive impairment in participants using a joystick to stay upright and avoid crashing while riding in a motion simulator programmed to topple as an inverted pendulum away from an upright direction of balance about two axes (2D) relative to 1D. The 2D test configuration provided participants with full vestibular/somatosensory cues about tilt relative to gravity. The 2D impairment was a) only marginally mitigated by vision relative to blindfolded conditions, and b) worse than a 1D decrement in a 0-g analog condition which precluded gravitational tilt cues by having participants balance in the horizontal roll plane where they were always 90 degrees from the vertical. With practice in the upright 2D balance task over two test days, the deficit declined nearly to the upright 1D level of proficiency. The outsized effect of expanding self-balancing from 1D to 2D and the surprisingly insubstantial visual recovery justify reconsideration of prevailing servo-like models and methods for assessing risk and preventing loss of life from aircraft mishaps, falls, and other tasks involving guidance of an unstable plant. As an alternative explanation, we expand a serial motor decision-making model originally developed for 1D balancing.

## Introduction

Predicting sensorimotor capability and risk in operational conditions from laboratory tests remains a difficult challenge. This is nowhere truer than for prediction and amelioration of falling (CDC 2026; Okoye et al. 2023; Eckstrom et al. 2024) and flight mishaps (Dixon et al. 2019; Roques et al. 2025) – adverse events which occur sporadically in multi-dimensional, multi-sensory tasks that are executed fluently most of the time. Bipedal stance though more complex than an inverted pendulum has been fruitfully framed as self-control of idealized 2-dimensional (2D) multi-link inverted pendulum biomechanics (Gurfinkel and Osovets 1972; Winter et al. 1996) employing a range of sensory inputs. The inverted pendulum model has proven applicable for both fore-aft and lateral sway despite their structural differences, leading to sensorimotor standards for evaluating and remediating fall risk which focus on muscle strength, multisensory acuity, and reflexes, though cognitive factors are beginning to be recognized (Drootin 2011). Helicopter piloting is a high-risk, 3D task, especially in low vision and bad weather conditions (Shekhar and Blumen 2023). Risks for hovering mishaps include the minimal auto-stabilization of helicopters in some axes, pilot susceptibility to illusory self-rotation when controlling azimuth without gravitational cues (Rupert et al. 2023), and pilot-induced command reversals in horizontal plane maneuvering (Tribukait et al. 2016). The inverted pendulum is only a partial analog of a helicopter but it preserves the fundamental general challenge faced by pilots of controlling an unstable plant.

Extending laboratory spatial tasks from 1D to progressively more realistic 2D or 3D conditions commonly reveals graded (Jagacinski and Monk 1985; Glasauer et al. 1997; Barnes 2008; Fitze et al. 2023) and occasionaly super-additive (McRuer 1980; Sheridan 1992) performance deterioration. However, the multi-axis expectation for inverted pendulum balancing has never been tested. Adding veridical multi-sensory cueing to degraded uni-sensory conditions generally yields additive or super-additive sensorimotor performance improvements, (Allum et al. 1976; Brandt et al. 1977; Leblond et al. 2026, Stanford and Stein 2007), including for visual-vestibular interactions (Ferre et al. 2015), but this has never been assessed for self-balancing.

The most common paradigms for studying multi-dimensional and multi-sensory spatial orientation give participants a task of rejecting 1D pseudorandom stationary disturbances of their body or of the visual field and modeling the effects with optimized continuous or intermittent, linear or linearized servo-like processes (Zacharias and Young 1981; Goodworth and Peterka 2012; Wada 2021; Love et al. 2026). Such models are optimized by fitting parameters to adaptive multi-sensory fusion of noisy signals or noisy anisometric effectors (Soechting and Flanders 1989; Tweed 2007; Rosenberg et al. 2018). A less common paradigm assesses user-driven balancing of 1D inverted pendulum dynamics, for example maintaining the upright attitude of an object (Cluff et al. 2011; Cabrera and Milton 2012; Morasso et al. 2019), one’s own body (Loram and Lakie 2002; Bottaro et al. 2005; Lakie and Loram 2006), or a computer-animated visual inverted pendulum (Zgonnikov et al. 2014). Self-driven balancing tasks are realistic and informative because they challenge participants to avoid definitive adverse events (crashes, falls) which are absent, un-defined or arbitrarily defined in tasks requiring the nulling of unpredictable, stationary disturbance stimuli. Our particular program of 1D self- and visual balancing studies differs from other balancing paradigms because it is designed to decouple loading of the manipulandum (e.g., legs) from the state of the balanced plant (e.g., body), thereby shunting peripheral servo-like factors (e.g., spinal and propriospinal reflexes and brainstem reflex coordination). Our method involves measuring how individuals seated in a motorized multi-axis rotating system (MARS) programmed to simulate inverted pendulum dynamics can stabilize their attitude by manipulating a joystick (Panic et al. 2015, 2017; Vimal et al. 2017, 2023; Wang et al. 2022; Mannan et al. 2024). While the MARS topples leftward or rightward, the passenger tries to stay at the vertical direction of balance (DOB) and to avoid “fall” boundaries. The MARS chair carries the joystick along with the participant, producing no deflection or force feedback on the hand. Consequently, no reflex-like manual righting is elicited. This shunting of reflex-like or servo-like processes enforces a reliance on high-level processes. We hypothesized that such high-level processes ordinarily contribute, along with spinal and brainstem righting mechanisms to all self-balancing, but they have never been isolated for 1D or 2D balancing.

The collective results gathered from previous MARS studies implicate central decision-like balancing processes in two ways. First, manual joystick commands are predominantly discrete and pulsatile rather than smooth and proportional (see Fig. 2), and about 10% of commands are frankly destabilizing in that they accelerate rather than retard pendular motion toward a fall. Such discrete, destabilizing commands are inconsistent with inflexible servo-like, reflex-like processes. The sudden destabilizing commands observed in MARS experiments are reminiscent of mishaps where pilots in off-nominal states of real and simulated flight made command actions that worsened the situation (Belcastro and Foster 2010; BEA 2012; Williams et al. 2018). Second, the probability of destabilizing manual commands decreases as a logistic function of diminishing time for the MARS to impact a fall boundary (Park et al. 2024), which is characteristic of decision errors made under time pressure.

Here, we conducted the first two experiments extending the study of self-balancing to multi-dimensional and multi-modal conditions., excluding reflex-like righting, with an emphasis on measurement of achieved and perceived plant (self) kinematics as well as manual command errors. In Experiment 1, we aimed to assess the magnitude of performance deterioration in acute 2D relative to 1D self-balancing, as well as the role of vision in mitigating decrements. We found super-additive 2D impairments weakly mitigated by vision, which are implausible results of current servo-like multi-dimensional, multi-sensory models but are compatible with an alternate high-level logistic motor decision model for which we provide post-hoc explanations. Experiment 1 also contradicted the common observation that many 2D or 3D tasks, including postural stance and piloting an aircraft, are performed fluently and benefit greatly from vision, yet systematic studies of how these skills developed de novo are incomplete. Therefore, Experiment 2 studied extended exposure to the 2D balancing task with and without vision. Participants showed rapid learning and little visual contribution, which we assert provides a new perspective on multi-dimensional, multi-sensory balancing as a fragile albeit learnable skill.

## Materials and methods

*Participants*: Twelve individuals (5 female, 7 males; 28 ± 11 years old were recruited for Experiment 1 and fifteen (6 female, 9 male, 24 ± 7 years old) for Experiment 2. Participants had with no task-relevant sensory and motor deficits or prior MARS experience were selected. All signed an informed consent form approved by the Brandeis Institutional Review Board.

*Apparatus*: Participants were confined in the MARS device simulating an inverted pendulum toppling away from a programmed direction of balancing (DOB) in an upright or horizontal plane. Participants deflected a Logitech Freedom 2.4 cordless joystick mounted on the MARS to avoid “crash” boundaries flanking the DOB at ±53° and to stay near the DOB (0°) with as little oscillation as possible. The full dynamics for the human-driven MARS pendular system around each axis were governed by $\ddot{\theta } = k_{P} \sin \theta + k_{J} J$, where $\ddot{\theta }$ and *θ * are MARS angular acceleration and position, *k*_*P*_ is the MARS intrinsic toppling rate, *J* is joystick deflection normalized to ±1 (actual range ±30°), and *k*_*J*_ is joystick gain. The sign of ${k}_{J}J$ implies that deflecting the joystick accelerated the MARS in that direction. Each axis was controlled and recorded individually, and a fall was recorded if the crash limits were exceeded in either axis. The selected toppling rate, *k*_*P*_, translates to a 2.6 s duration free fall with no joystick deflection from 1° to the 53° crash boundary. In previous MARS studies (Panic et al. 2017; Vimal et al. 2017), this fall rate had proven challenging but not frustrating. For safety and comfort, we limited the maximum angular acceleration to ±300°/s^2^ and the maximum angular velocity to ±180°/s. The joystick gain, *k*_*J*_, was calibrated to accelerate the MARS in the same direction as joystick deflection and to hold it in static equilibrium at the crash boundary at full joystick deflection magnitude. If a crash boundary was exceeded, MARS toppling was suspended and joystick inputs disabled and resumed after an automatic MARS reset back to the DOB followed by an audible “begin” notice. Mounting the joystick base on the chair arm eliminated haptic feedback cues correlated with MARS state. A “kill switch” on the left armrest could rapidly stop the experiment but it was never used.

**Fig. 1:**
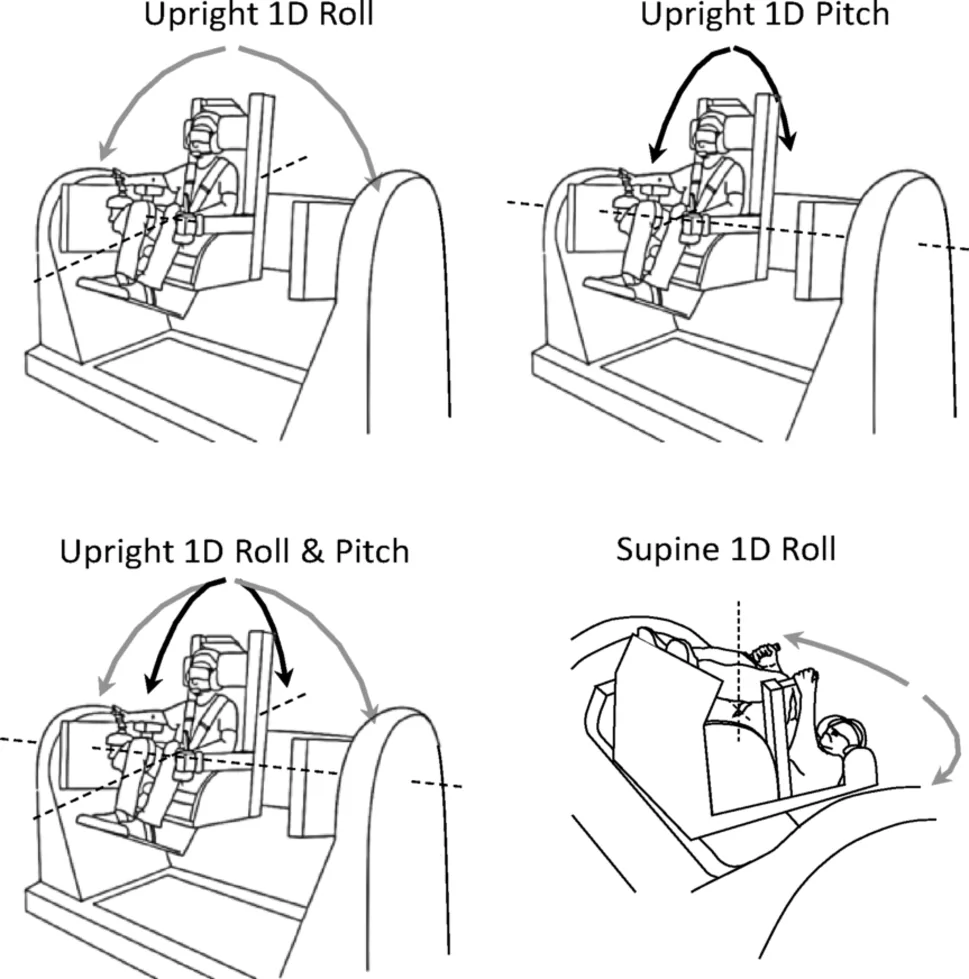
Illustration of the conditions included in Experiment 1, labelled consistently with text. The participant is shown wearing a blindfold and communication headset. Black and gray arrows represent MARS pitch and roll, respectively

*Procedure*: Experiment 1 was primarily designed to assess the magnitude of performance deterioration in acute 2D relative to 1D self-balancing when task-relevant gravity cues were available, as well as the role of vision in mitigating 2D decrements. It also compared the 2D decrements to performance in a previously validated (Vimal et al. 2019, 2025) 1D spaceflight analog condition which eliminates task-relevant gravitational cues. Participants underwent a 1-h protocol consisting of 20 trials organized into 1 block of practice trials and 3 blocks of 6 trials where data were collected. Blocks 2–4 each contained one of three MARS balancing conditions per participant, repeated three times with eyes closed (EC) and eyes open (EO) alternating. See Table 1. The MARS balancing configurations included: 1) either Upright 1D Roll or Upright 1D Pitch (subsets of n = 6 per condition), 2) Upright 2D Roll & Pitch (n = 12), and 3) Supine 1D Roll (n = 12), as illustrated in Fig. 1. In Block 1, each participant received four familiarization trials with eyes closed in the same MARS balancing mode as the Upright 1D (Roll or Pitch) trials to which they were assigned. Upright 1D and 2D balancing EC entail MARS tilt relative to the gravitational vertical encoded by otolith and slowly adapting somatic mechanoreceptor signals and inertial angular oscillations encoded by semicircular canals and rapidly adapting mechanoreceptor signals. Supine 1D Roll balancing was included as a benchmark because it is a documented space flight 0-g analog which precludes task-relevant gravity tilt cues by having participants balance in the horizontal roll plane where they were always 90 degrees from the vertical, which induces serious performance decrements (Vimal et al. 2019, 2025).

**Table 1:** Designs for Experiments 1 and 2

Participant	Practice	Data collection		
	Block 1 (4 trials)	Block 2 (6 trials)	Block 3 (6 trials)	Block 4 (6 trials)
*Experiment 1*				
1–6	Upright 1D Roll, EC	Upright 1D Roll ×3, each with EO, EC alternating	Upright 2D Roll & Pitch ×3, each with EO, EC alternating	Supine 1D Roll ×3, each with EO, EC alternating
7–12	1D Upright Pitch EC	Upright 1D Pitch ×3, Each with EO and EC alternating	Upright 2D Roll & Pitch ×3, each with EO and EC alternating	Supine 1D Roll ×3, each with EO and EC alternating
		Order of balance configurations across Blocks 2–4 counterbalanced; Order of alternating visual conditions within blocks counterbalanced		
*Experiment 2*				
Participant	Practice	Data Collection		
		Day 1, Blocks 1–5 (4 trials/block)	Day 2, Blocks 6–10 (4 trials/block)	
Odd (n=8)	None	Upright 2D Roll & Pitch ×2, each with EO and EC alternating		
Even (n=7)		Upright 2D Roll & Pitch ×2, each with EC and EC alternating

Before data collection, participants were informed that the MARS behaved like an inverted pendulum and watched videos of another person in the MARS using the joystick to balance in all three conditions they would later experience. Then they gave informed consent and were secured in the MARS using a lap belt, a five-point safety harness, side torso panels, and a U-shaped, foam-lined head restraint. They inserted earplugs and donned a headset which fit into in the contoured foam headrest, which actively cancelled ambient sound and also played white noise interrupted by voice commands (trial start/end, reset start/end). They wore a blindfold and the room lights were also turned off during the eyes covered (EC) trials, and they could raise the blindfold for eyes open (EO) trials allowing an unrestricted view of the fully illuminated experimental chamber. With EO, participants in the Upright 1D and 2D conditions saw a bench at which the operator was seated which supported two elaborately wired equipment cabinets and two computers as well as a wall in the background that had a blackboard and portions of the floor and ceiling when pitch angle was large, at distances ranging 1.5–2.5 m. In Supine 1D Roll, participants looked up the tiled ceiling above a cross-beam spanning the trunnion pedestals for the MARS outer gimbal axis on which various small electronic units were mounted, at distances ranging from 1 to 2.5 m. Each trial consisted of 100 s of total balance excluding crash reset time. We stopped trials with many crashes at 120 s to avoid taxing the participants, which yielded less than 100 s of active balancing in 37% of trials. Trial truncation occurred predominantly in the difficult Upright 2D and Supine 1D Roll conditions, but this did not bias comparisons to the easier Upright 1D conditions because the duration of truncated trials was only slightly reduced to 91 ± 3 s and previous unpublished results had shown that the first and last 50 s of trials do not differ. There were 1-min rests between trials with the participant in an upright attitude, and longer breaks between blocks for collection of subjective ratings. At the end of every block, participants rated two aspects of the balancing task for each MARS configuration: Uncertainty about perceived angular position, where 0 was ‘not confused’ and 10 was ‘very confused (PosConfu); The level of attention required, where 0 was ‘required no attention, I could easily think about other things’ and 10 was ‘it took my complete concentration and attention’ (Atten). After Block 4, participants rated how much vision had helped in each MARS configuration, where 0 was ‘vision was not helpful’ and 10 was ‘vision was very helpful, I wouldn’t be able to balance without it’ (VisAid).

Table 2 here.

**Table 2:** Statistical results for experiment 1

Dependent variable	Analyses of variance					^‡^Paired t tests between axes					
	Factor	df	F	p	Effect size (r^2^)	Axes compared	% Change	^$^ df	t	& _p_	Effect size (r^2^)
^#^RMS_Roll_	Axis	2, 10	26.229	0.00011	0.29	Upright 1D Roll versus Upright 2D	+ 86	5	4.501	0.01918	0.80
	Vision	1, 5	150.925	0.00006	0.25	Upright 1D Roll versus Supine 1D Roll	+ 146	5	6.097	0.00515	0.88
	Axis*Vision	2, 10	26.229	0.00011	0.21	Upright 2D versus Supine1D Roll	+ 33	5	2.694	0.12924	0.59
^$^RMS_Pitch_	Axis	1, 5	36.450	0.00180	0.33	Upright 1D Pitch versus Upright 2D	+ 47	5	2.806	0.03775	0.61
	Vision	1, 5	11.880	0.01830	0.24	Upright 1D Pitch versus Supine 1D Roll	NA	NA	NA	NA	NA
	Axis*Vision	1, 5	0.223	0.65684	0.00	Upright 2D versus Supine1D Roll	NA	NA	NA	NA	NA
^*^Crash	Axis	2, 20	18.773	0.00003	0.42	Upright 1D versus Upright 2D	+ 584	11	5.380	0.00067	0.72
	Vision	1, 10	8.014	0.01783	0.04	Upright 1D versus Supine 1D Roll	+ 322	11	4.102	0.00526	0.60
	Axis*Vision	1, 10	1.309	0.27914	0.01	Upright 2D versus Supine1D Roll	− 38	11	2.144	0.16555	0.29
^*^%Destab	Axis	2, 20	56.702	<0.00001	0.38	Upright 1D versus Upright 2D	+ 110	11	8.470	0.00001	0.87
	Vision	1, 10	52.327	0.00003	0.17	Upright 1D versus Supine 1D Roll	+ 90	11	5.125	0.00099	0.70
	Axis*Vision	1, 10	0.028	0.87114	0.00	Upright 2D versus Supine1D Roll	− 9	11	1.210	0.75502	0.12
^*^PosConfu	Axis	2, 20	13.873	0.00017	0.15	Upright 1D versus Upright 2D	+ 144	11	4.690	0.00198	0.67
	Vision	1, 10	64.918	0.00001	0.43	Upright 1D versus Supine 1D Roll	+ 240	11	7.416	0.00004	0.83
	Axis*Vision	1, 10	0.015	0.90526	0.00	Upright 2D versus Supine1D Roll	+ 39	11	2.636	0.06950	0.39
^*^Atten	Axis	2, 20	31.659	< 0.00001	0.41	Upright 1D versus Upright 2D	+ 107	11	6.148	0.00022	0.77
	Vision	1, 10	67.697	0.00001	0.16	Upright 1D versus Supine 1D Roll	+ 77	11	3.513	0.01458	0.53
	Axis*Vision	1, 10	1.698	0.22177	0.00	Upright 2D versus Supine1D Roll	− 15	11	1.761	0.31775	0.22
^*,^^VisAid	Axis	2, 20	30.065	0.00000	0.09	Upright 1D versus Upright 2D	+ 48	11	2.237	0.14088	0.31
	Vision	NA	NA	NA	NA	Upright 1D versus Supine 1D Roll	+ 135	11	11.192	<0.00001	0.92
	Axis*Vision	NA	NA	NA	NA	Upright 2D versus Supine1D Roll	+ 135	11	4.772	0.00174	0.67

^#^RMS_Roll_ is compared across all 3 conditions, within the group (n=6) tested in Upright 1D Roll

^$^RMS_Pitch_ is compared only for Upright 1D and Upright 2D, within the group (n=6) tested in 1D Upright Roll. (RMS_Pitch_ is null in Supine 1D Roll)

^*^%Crash, %Destab, PosConfu, Atten and VisAid are compared across all three conditions, for both groups

^^^VisAid is only measured in eyes open conditions, so the effect of Vision cannot be evaluated

^‡^All t tests are performed for eyes closed conditions, except for VisAid which was only measured for eyes open conditions

^&^*p* values Bonferroni corrected for 3 family-wise comparisons per variable

Experiment 2 studied extended exposure to the 2D balancing task with and without vision. The large deficits found in Experiment 1 are at odds with the fluent 2D behavior of healthy adults maintaining postural balance and of experts piloting aircraft; No systematic knowledge exists about the early time course of acquisition of such skills which require controlling an unstable plant. The goal was to characterize the rate of learning the primary task—avoiding adverse events—and to identify the underlying sensorimotor adjustments. The initial trials of extended practice assess replicability of the acute 2D deficits found in Experiment 1, and subsequent trials would assess the time course of learning to balance in 2D and reveal the sensorimotor processes underlying it, including the role of vision. The user-driven MARS dynamics In Experiment 2 were identical to Experiment 1. For familiarization, participants listened to a description of the MARS inverted pendulum behavior and watched a video of another person performing Upright 2D Roll & Pitch balancing, but they did not receive the real 1D practice which had been allowed in Experiment 1; Instead they began performing Upright 2D Roll & Pitch balancing immediately after giving informed consent. Each test day was organized as 5 blocks of 4 100-s trials including alternating EC and EO conditions whose order was counterbalanced across participants (see Table 1). Subjective ratings of how much vision helped (VisAid) were done after every block instead of at the end of the session as in Experiment 1.

**Fig. 2:**
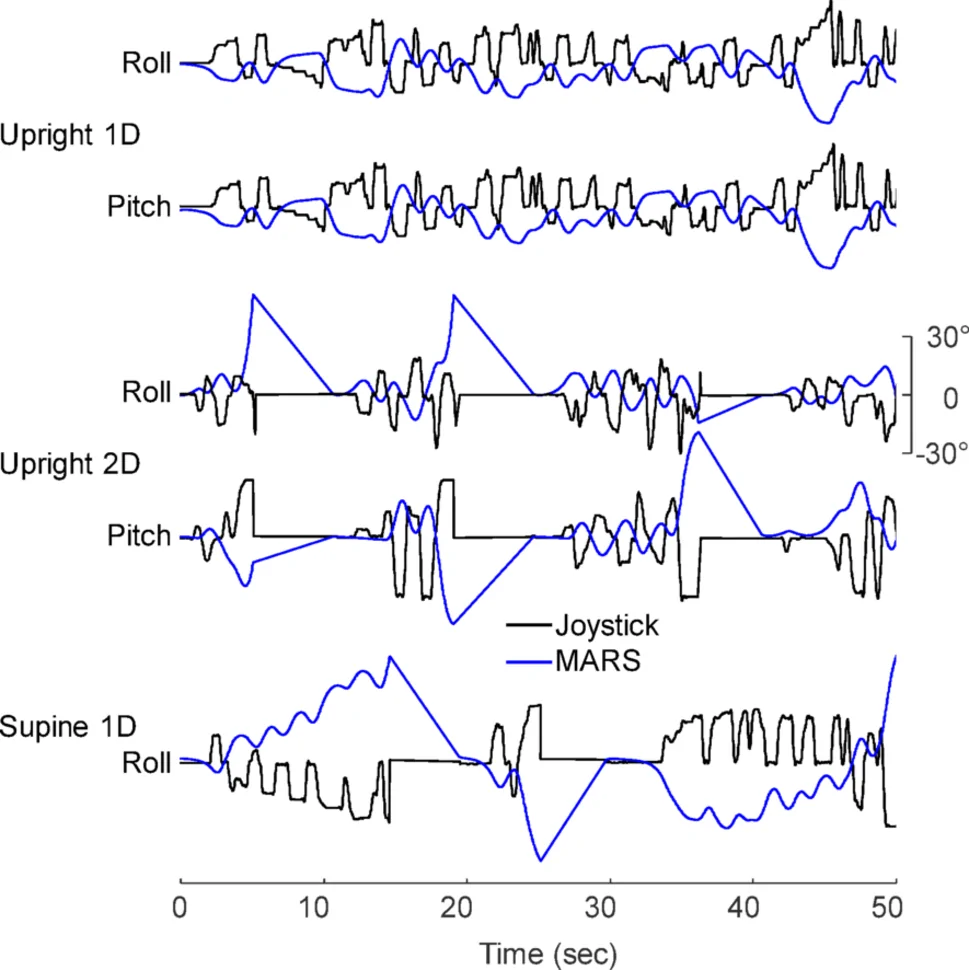
Typical time series of MARS and joystick angular positions for 50 s periods of each of the four experimental conditions. The Upright 1D Roll, Upright 2D Roll & Pitch, and Supine 1D Roll conditions are all from the same participant; The Upright 1D Pitch example is from a different participant. No falls occur in the Upright 1D Roll and Upright 1D Pitch conditions, three falls in the Upright 2D condition, and two in Supine 1D Roll. Falls were defined when the MARS reached ±53° in either axis, after which it returned automatically toward upright at a constant rate and the joystick was disabled until the MARS was upright. Joystick commands were predominantly discrete and pulsatile, as had been seen in all previous MARS experiments

*Data reduction*: In Experiment 1, the 1D practice block was not analyzed. For both experiments, MARS roll and pitch angle (DOB=0°) and velocity were sampled, at 48 ± 3 Hz and filtered (Butterworth zero-phase 5-pole low-pass, cutoff frequency of 5 Hz). Trial performance was quantified in terms of crashes per minute (Crash), MARS positional oscillation in roll and pitch (RMS_Roll_, RMS_Pitch_), and the percentage of samples with destabilizing joystick deflections in the same roll or pitch direction as MARS displacement and velocity (%Destab). Each participant’s performance was averaged across all trials and blocks per condition. Figure 2 presents time series of MARS angle and joystick deflection for typical trials, with annotations on data reduction. Statistical analyses of all variables (RMS_Roll_, RMS_Pitch,_ Crash, %Destab, PosConfu, Atten, VisAid) across participants were performed with the MATLAB *manova*, *ranova*, *mauchly*, and *grpstats* functions.

## Results

### Experiment 1

**Fig. 3:**
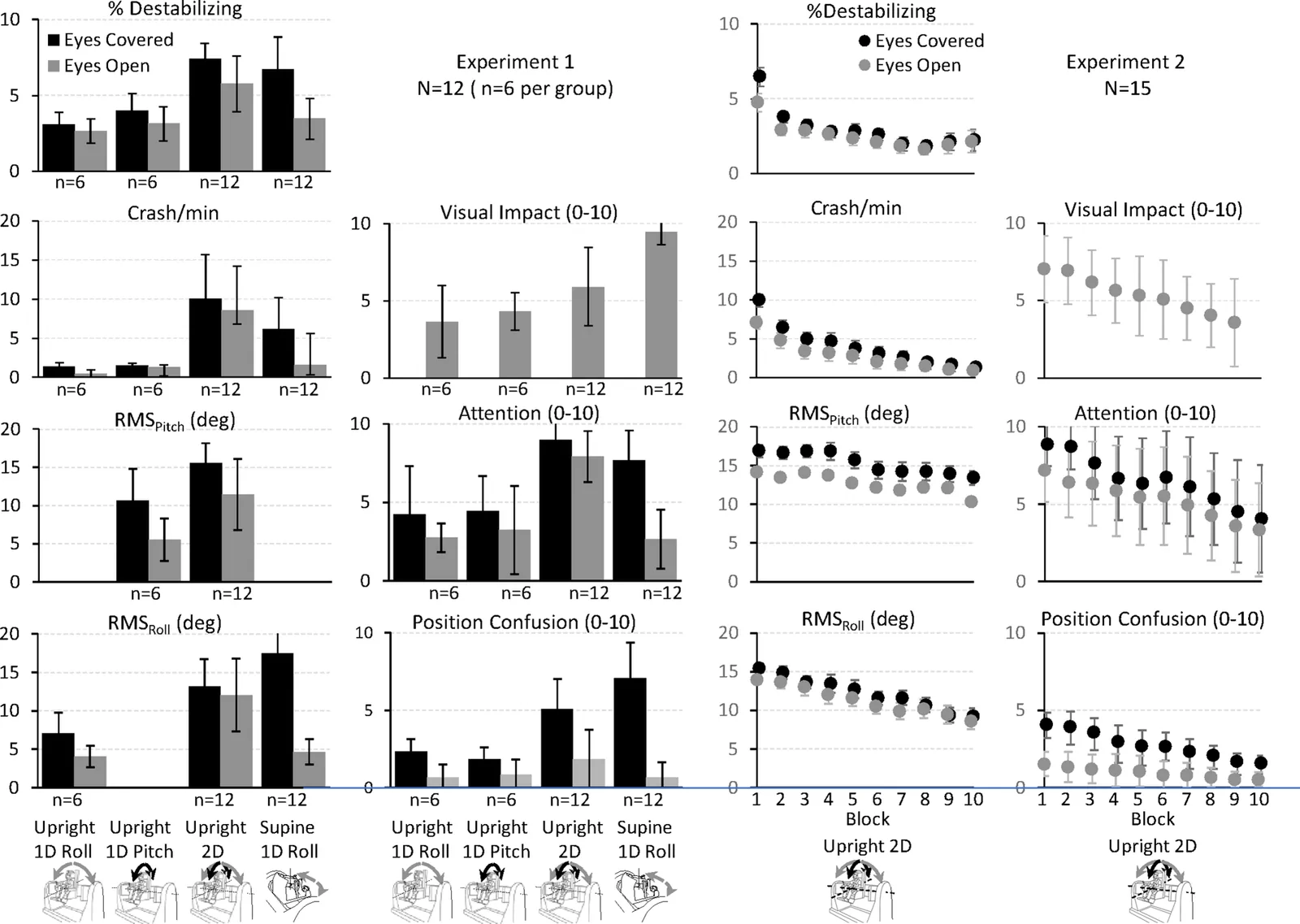
The two left columns summarize objective performance and subjective self-ratings during acute exposure in Experiment 1 during four MARS balance configurations labelled and illustrated at the bottom. Subgroups of 6 participants performed either Upright 1D Roll or Upright 1D Pitch and are plotted separately, and all twelve participants performed Upright 2D Roll & Pitch and Supine 1D Roll, which are plotted jointly. The right two columns summarizes Experiment 2 in which participants performed repeated Upright 2D trials, with Blocks 1–5 and 6–10 on consecutive days. All error bars represent standard deviations across participants after trial repetitions had been averaged

The left half of Fig. 3 illustrates the results for Experiment 1. Preliminary ANOVAs comparing the subgroups who performed Upright 1D Roll or Upright 1D Pitch showed no significant differences between RMS_Roll_ and RMS_Pitch_, respectively, and other variables, with small effect sizes ranging from r^2^ = 0.005 to r^2^ = 0.200. However, the observed powers of the subgroup differences were too low (5–6.9%, GPower v3.1.9.7) for the negative results to be conclusive. Thus, the Upright 1D Roll and Upright 1D Pitch subgroups were plotted separately in Fig. 3, to allow visualization of both the small decrement in Pitch relative to Roll and their individual comparisons to Upright 2D Roll & Pitch and Supine 1D balancing, but statistical analyses (ANOVAs) of all variables maintained the between-participant effect of subgroup (Upright Roll or Pitch) as well as the within-participant effects of vision (EO and EC) and the three balance conditions (Upright 1D, Upright 2D and Supine 1D). See Table 2, left side. RMS_Roll_ and RMS_Pitch_ were measurable only in the corresponding Upright 1D conditions, so separate ANOVAs on the two within-participant factors (balancing condition and vision) were conducted for each subgroup thereby preventing evaluation of group differences. Moreover, RMS_Pitch_ was null in the Supine 1D Roll condition, so only two balancing conditions—Upright 1D Pitch and Upright 2D Roll & Pitch—could be compared. VisAid was only measured in the EO condition, so the effect of vision could not be evaluated. ANOVAs showed no significant main effects or interactions of group identity for variables measured in both subgroups (Crash, %Destab, PosConfu, Atten, VisAid). However, the balancing condition had a significant main effect (controlling for subgroup) on all variables. The effects of balancing condition on MARS performance variables (RMS_Roll_, RMS_Pitch_, Crash, %Destab) were highly significant, *p* = 0.00180 for RMS_Pitch_ to *p* < 0.00001 for %Destab, with moderate to large effect sizes ranging from r^2^ = 0.288 for RMS_Roll_ to r^2^ = 0.415 for Crash. Vision significantly improved all variables for which it could be evaluated (VisAid not eligible), *p* = 0.0180 to *p* < 0.00001, with effect sizes ranging from r^2^ = 0.044 to r^2^ = 0.426. The interaction of balance condition and vision was only significant for RMS_Roll_, where vision improved Supine 1D Roll more than Upright 1D and 2D balancing. However, Fig. 3 shows a general interaction pattern where vision mitigates performance of the large Supine 1D Roll decrements more than the equally large Upright dual axis decrements. The EC to EO improvements in the Upright 2D Roll and Supine 1D Roll conditions averaged across RMS_Roll_, Crash, and %Destab were 15.1%, and 65.2%, respectively.

The patterns of significant differences across balancing conditions were evaluated with post-hoc paired t tests, within subgroups. See Table 2, right side. Only EC performance was assessed because multi-axis decrements were expected to be maximal under degraded visual conditions. Upright 2D Roll & Pitch was compared to Upright 1D Roll or Pitch and to Supine 1D Roll where possible (RMS_Pitch_ not eligible), within subgroups; The former evaluated the multi-axis deficit and the later compared the deficit in dual axis balancing with gravity cues relative to the single axis deficit in the 0 g analog lacking gravity cues. Upright 1D Roll balancing was also compared to Supine 1D roll, to replicate the previously documented deficit in controlling unstable dynamics in 0 g analog conditions. *P* values were Bonferroni-corrected for the number of comparisons per variable (up to 3 comparisons). Dual-axis MARS balancing was significantly worse than single axis when gravity cues were available (Upright 1D vs Upright 2D), for every variable, ranging from *p* = 0.038 for RMS_Pitch_ to *p* < 0.00001 for VisAid; The effect sizes for these comparisons were very large, average r^2^ = 0.595. The roughly 500% magnitude increase in dual axis crash frequency outpaced the average 90% increases for all other variables combined. The same pattern of decrements was found comparing in Supine 1D Roll where gravity cues were eliminated to Upright single-axis balancing with gravity cues. Those t tests were highly significant with very large r^2^ values, and, again, crash frequency outpaced all other variables in its percent increase in magnitude. Pairwise comparisons between dual axis balancing with gravity cues (Upright 2D Roll & Pitch) and single axis balancing without gravity cues (Supine 1D Roll) were only significant for the subjective assistance from vision (VisAid), which was lower in the dual axis condition. Overall, the results represent super-additive rather than incremental decrements in 2D relative to 1D Upright balancing, including an increase in subjective attentional demand from moderate in 1D to maximal in 2D. The 1D to 2D decrements were comparable to the effects of removing gravity cues for 1D balancing. Full natural sight of the upright stationary environment marginally reduced 2D balancing decrements, objectively and subjectively, but it did mitigate the decrements induced by elimination of gravity cues.

### Experiment 2

The fivefold 2D decrement and ineffectiveness of vision for mitigating them found in Experiment 1 were surprising in light of common observations that 2D or 3D unstable tasks such as flying an aircraft or helicopter, driving a boat or car, and maintaining bipedal stance can be accomplished expertly but suffer under visual degradation. However, everyday observation might overlook the role of learning and its fragility in the mastery of multi-axis unstable tasks with fundamentally super-additive multi-dimensional difficulty. In other words, expert and novice performance may look similar but involve different mechanisms. Experiment 1 had included no training with the 2D task prior to a mere three 2D trials per visual condition, so Experiment 2 assessed performance in just the 2D Upright Roll & Pitch balancing task over 40 trials split across two consecutive days. The goal was to characterize the rate of learning the primary task—avoiding adverse events—and to identify the underlying sensorimotor adjustments.

The right half of Fig. 3 illustrates the average performance improvement in Upright 2D MARS balancing across repeated EC and EO blocks (2 100-s trials per visual condition per block) in Experiment 2. Block 1 values for all dependent measures replicated the 2D decrements seen in Experiment 1 (right side of Fig. 3), including the minimal effect of vision and high attentional load. The ANOVAs in Table 3 show that 2D performance decrements for all dependent measures abated significantly across blocks, *p* > 0.00001 for all variables. The t tests on the right side of Table 3 show that for EC conditions Block 10 on Day 2 was significantly better than the initial Block on Day 1 for all measures. From Block 1 to 10, the 81% reduction in crash frequency magnitude and the 29% average reduction in RMS_Pitch_ and RMS_Roll_ were the largest and smallest improvements, respectively. Vision significantly improved 2D performance as well but its effect was contingent on block for Crash, %Destab, PosConfu, and Atten; In Block 1, EO significantly improved all variables except RMS_Roll_ relative to EC, however, in Block 10 EO produced no significant improvement in three variables—RMS_Roll_ again, Crash, %Destab. The average effect size for the visual mitigation across all variables was 0.69 in Block 1 and 0.35 in Block 10.

**Table 3:** Statistical results for Experiment 2

Dependent variable	Analyses of variance					^‡^Paired t tests					
	Factor	df	F	*p*	Effect size (r^2^)	Axes compared	%Change	^$^df	t	&_p_	Effect size (r^2^)
RMS_Roll_	Block	9, 126	17.080	< 0.00001	0.19	Block 1 versus Block 10, Eyes closed	− 40	11	13.811	0.00011	0.95
	Vision	1, 14	6.645	0.02190	0.01	Eyes closed versus Eyes open, Block 1	− 11	11	2.429	0.17834	0.35
	Block*Vision	9, 126	1.101	0.36677	0.00	Eyes closed versus Eyes open, Block 10	− 7	11	1.625	0.49497	0.19
RMS_Pitch_	Vision	9, 126	5.821	< 0.00001	0.11	Block 1 versus Block 10, Eyes closed	− 21	11	3.732	0.04064	0.56
	Block	1, 14	15.139	0.00163	0.12	Eyes closed versus Eyes open, Block 1	− 20	11	11.054	0.00032	0.92
	Block*Vision	9, 126	0.636	0.76439	0.00	Eyes closed versus Eyes open, Block 10	− 30	11	7.026	0.00270	0.82
Crash	Block	9, 126	32.281	< 0.00001	0.26	Block 1 versus Block 10, Eyes closed	− 87	11	87.305	<0.00001	1.00
	Vision	1, 14	11.455	0.00445	0.02	Eyes closed versus Eyes open, Block 1	− 41	11	13.216	0.00013	0.94
	Block*Vision	9, 126	4.147	0.00011	0.01	Eyes closed versus Eyes open, Block 10	− 44	11	1.191	0.86171	0.11
%Destab	Block	9, 126	13.514	< 0.00001	0.22	Block 1 versus Block 10, Eyes closed	− 65	11	9.188	0.00077	0.88
	Vision	1, 14	8.260	0.01225	0.01	Eyes closed versus Eyes open, Block 1	− 36	11	7.033	0.00269	0.82
	Block*Vision	9, 126	2.418	0.01444	0.01	Eyes closed versus Eyes open, Block 10	− 5	11	0.890	1.00000	0.07
PosConfu	Block	9, 126	8.522	< 0.00001	0.06	Block 1 versus Block 10, Eyes closed	− 61	11	10.406	0.00042	0.91
	Vision	1, 14	31.039	0.00007	0.16	Eyes closed versus Eyes open, Block 1	− 62	11	9.426	0.00068	0.89
	Block*Vision	9, 126	4.712	0.00002	0.01	Eyes closed versus Eyes open, Block 10	− 67	11	4.893	0.01351	0.69
Atten	Block	9, 126	14.616	< 0.00001	0.19	Block 1 versus Block 10, Eyes closed	− 54	11	11.033	0.00032	0.92
	Vision	1, 14	41.017	0.00002	0.04	Eyes closed versus Eyes open, Block 1	− 19	11	11.728	0.00024	0.93
	Block*Vision	9, 126	3.719	0.00036	0.01	Eyes closed versus Eyes open, Block 10	− 18	11	3.988	0.03133	0.59
^^^ VisAid	Block	9, 126	10.123	< 0.00001	0.04	Block 1 versus Block 10, Eyes *open*	− 56	11	6.363	0.00425	0.79
	Vision	NA	NA	NA	NA	Eyes closed versus Eyes open, Block 1	NA	NA	NA	NA	NA
	Block*Vision	NA	NA	NA	NA	Eyes closed versus Eyes open, Block 10	NA	NA	NA	NA	NA

^^^VisAid is only measured in eyes open conditions, so the effects of Vision cannot be evaluated

^‡^Comparisons of Block 1 versus Block 10 are performed for eyes closed conditions, except for VisAId which was only measured for eyes open conditions

^&^*p* values Bonferroni corrected for 3 family-wise comparisons per variable

## Discussion

These extreme patterns of 2D balancing degradation are surprising and meaningful in five respects. *First*, they are the first results from a study comparing 1D and 2D self-balancing in a paradigm which minimizes reflex-like self-correction, in a task with an unstable plant. The degradations observed here are an order of magnitude larger than deficits seen in a broad survey of multi-dimensional skill assessments (Jagacinski and Monk 1985; Glasauer et al. 1997; Barnes 2008; Fitze et al. 2023), though they are compatible with a small set of unexplained cases where multi-dimensional manual guidance evoked large deficits (McRuer 1980; Sheridan 1992). Peripheral encoding and actuation are not responsible for our 2D decrements because the distributions of head (MARS) and hand (joystick) kinematics did not exceed either the distributions in the 1D task nor known peripheral limits for vestibular orientation encoding (Mohammadi et al. 2024) or wrist actuation (Charles and Hogan 2011). We propose that a central balancing process is responsible, as described below. *Second*, such a cognitive alternative to proportional servo-like balancing mechanisms for explaining all the results is reinforced because our methodology rules out biomechanical and reflex-like righting by decorrelating joystick and MARS state. Such a central mechanism could work in concert with reflex-like righting in natural or laboratory tasks where the effector feeds back information about the state of the plant, such as the feet and legs do during weight-bearing postural sway. However, natural man–machine interactions and our MARS task which remove or degrade reflection of kinetic and kinematic feedback about the inverted pendulum or other unstable plant must rely on more flexible central mechanisms. *Third*, we replicated the finding that Supine Roll is a partial analog of operational space flight because it degrades performance by depriving users of a task-relevant gravitational reference (Vimal et al. 2017). Although an inverted pendulum is physically undefined in 0 g, other sources of unstable dynamics may exist in 0 g. *Therefore*, it is surprising that we found extension from 1 to 2D in the presence task-relevant gravity cues caused deficits which equaled or exceeded 1D deficits where one of multiple task relevant cues were missing. (In Supine Roll, otolith cues provide no information about pendular displacement but semicircular canals (EC) and vision (EO) provide useful information.) The central balancing mechanism we propose below can explain this result which is a paradox for linear servo-like controllers modelling humans as linear ideal observers. *Fourth*, There is no current explanation for why we would observe that the deficits in blindfolded Upright 2D Roll & Pitch are only marginally mitigated by vision while Supine Roll balancing deficits are largely eliminated. Prevailing adaptive multi-sensory integration models predict that veridical vision rescues blindfolded Supine Roll balancing deficits by replacing missing vestibular/somatic cues about the gravitational upright (Zacharias and Young 1981; Goodworth and Peterka 2012; Wada 2021), but a different multi-sensory component is inherent in the central balancing process we outline below to explain the marginal visual remediation of 2D balance deficits. Extending the observation of preferential visual suppression of MARS Supine Roll over Upright 2D balancing to unstable piloting tasks suggests the practical implication that risky operational scenarios like controlling the heading and position of a hovering helicopter or spacecraft will suffer from *both* minimal gravity-cueing and an imperfect multi-dimensional balance controller which cannot be rescued by powerful visual cues. By extension, new multi-sensory integration models may be required to explain analogous forms of operational disorientation. For example, vision is not a fully effective countermeasure against 2D Roll & Pitch nor the somatogravic illusion responsible for aircraft crashes (Previc et al. 1992); By contrast, that vision mitigates Supine Roll deficits may be partially analogous to the fact that visual deprivation or augmentation is a powerful risk factor or countermeasure, respectively, in helicopter hovering (Shekhar and Blumen 2023). *Fifth*, the learning pattern observed for Upright 2D balancing illuminates rather than contradicts the popular consensus that humans are fully capable of multi-dimensional tasks like piloting and bipedalism. We propose that sporadic flight mishaps and falls reflect the properties of a CNS balancing mechanism which is overwhelmed and immune to visual rescue in novices, and is still attention-demanding and fragile after insufficiently understood adaptation acquired through practical experience.

One plausible candidate consistent with degradation of 2D self-balancing and analogous real-world tasks is an extension of the serial motor decision process we developed to account for age group differences in balancing a 1D visual inverted pendulum, alluded to in the Introduction (Park et al. 2024). In that study, we found a state-specific age difference, in which both groups performed identically when the pendulum approached the upright DOB but only the older adults showed a deficit when it approached a fall boundary. The probability of anti-corrective destabilizing joystick commands decreased and corrective commands increased reciprocally as a logistic function of the time to impact a fall boundary for both age groups, but older adults made more destabilizing commands and switched to corrective commands later. Neither pendulum kinematics nor joystick commands in the state where the pendulum approached the DOB correlated with fall frequency, but destabilizing commands correlated strongly with fall frequency when falls were imminent. The phenomenon of state-specific destabilizing commands logistically dependent on time pressure is problematic for servo-corrective automata but is characteristic of cognitive decision processes. It is not surprising that a deficient serial decision would increase the likelihood of falling when the time to contact is too brief for a correction but would not impact falling during approach to the upright (escaping from a fall boundary). Servo-like mechanisms neither distinguish escaping versus approaching a fall boundary nor permit logistic response-switching in the later. Our model of phase-dependent logistic decisions under time pressure is consistent with our observation that extending the MARS task from Upright 1D to Upright 2D in Experiment 1 caused the large changes in crash rate, destabilizing joystick commands, and attention but more modest changes in RMS displacement; In a similar vein, the improvement in 2D balancing with practice in Experiment 2 was manifest most strongly as reduction of crash rate, destabilizing joystick commands, and attention and more modestly in RMS displacement. Our model also admits the testable hypothesis that the flow of unpredictable visual information is too slow for making correct action decisions under extreme time pressure.

**Fig. 4:**
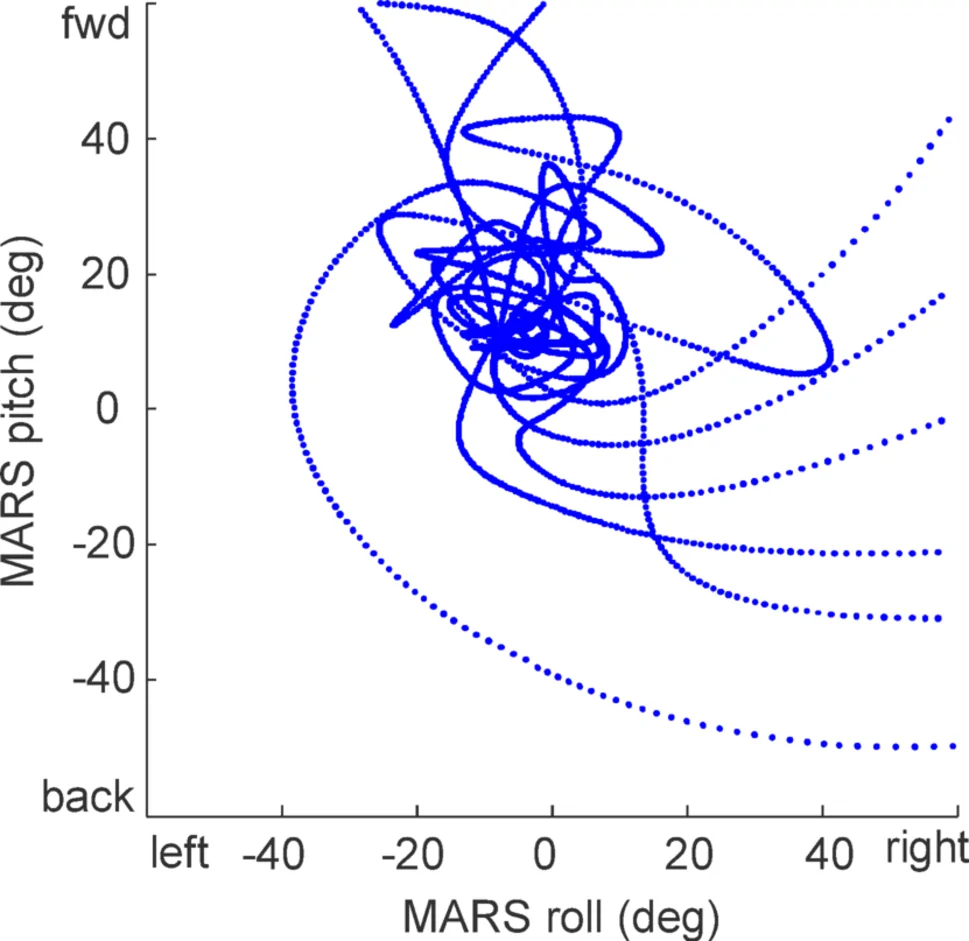
Typical plot of MARS roll and pitch in the 2D Roll & Pitch condition. Trajectories include circular revolutions around the DOB generating centripetal forces which require compensations beyond the component compensations for each cardinal axis displacement from the DOB

One testable hypothesis for expanding the 1D decision model to fit our MARS 2D self-balancing deficits is for the decision space to become super-additively complex. In MARS Upright 1D Roll or Upright Pitch, there are 2 choices of joystick deflection for countering the simulated gravitational field when a fall is imminent–left–right for roll or anterior–posterior for pitch. However, for 2D balancing there are more than the union of these four (2 per axis) independent alternatives. This could come about in several ways. First, the 2D pendulum revolves around the DOB rather than moving strictly in one or the other cardinal axis thereby generating a velocity-dependent centripetal acceleration in addition to the tilt-dependent gravitational acceleration See Fig. 4. This would generate super-additive 2D deficits if command decisions should address the gravitational accelerations common to 1D and 2D balancing but not the centripetal accelerations unique to 2D. The improvement with practice we observed could result from a remapping of 2D joystick commands onto the gravitational as well as centripetal accelerations. This is reminiscent of the rapid adaptation of arm, head, and leg movements to the large initial errors caused by Coriolis forces in a rotating artificial gravity environment (Lackner and DiZio 1994). A second hypothesis is combinatorial expansion of the decision space; In 1D the two possible states are escaping versus approaching the DOB, independently per axis, but for the 2D pendulum it is physically possible to simultaneously approach the DOB in one axis and to escape it in another axis, resulting in a super-additive number of decision states. Both post-hoc hypotheses are testable for explaining the surprising results. Testing these hypotheses will require a paradigm which differentiates them from servo-like physiological mechanisms which likely operate concurrently.

The traditional paradigm of observing responses to unpredictable stationary disturbances of 1D stimuli and then modeling the data with a servo-like automaton may contribute to understanding self-balancing up to a point, but it is not equipped to handle realistic multidimensional adverse events and is incapable of predicting them or prescribing training for surviving them. Our alternative balancing paradigm and decision model is a framework for overcoming those shortcomings. For example, it puts a premium on destabilizing commands that reduce the time window for recovery from falling or crashing and that can be monitored in real time, thereby providing an opportunity for real time mishap avoidance and training. Approaches analogous to ours which apply dynamical modeling to sufficiently complex tasks have advanced the understanding and engineering of other realistic multi-dimensional haptic skills and their neural bases (Bazzi and Sternad 2020; Sohn et al. 2020; Balasubramaniam et al. 2021), relative to classic optimization of models operating on surface-level dynamics. Furthermore, our framework raises the urgency of understanding the neural basis of motor decisions (Romo and Salinas 2001; Costello et al. 2013; McDougle and Hillman 2025), which is not traditionally considered relevant for understanding self-balancing. Our model of serial decisions contingent on the time to reach a crash boundary compels comparative analysis of the literature on perceptual-motor coordination of escaping looming threats, seeking prey, and avoiding environmental barriers (Lee 1976; Hecht et al. 2021; Leblond et al. 2026), as well as their neural bases (Frost and Sun 2004; Billington et al. 2011; Browning 2012; Baurès et al. 2021), for eradicating flight mishaps and falling.

## Conclusion

The major empirical results from both studies are: 1) *Large 2D deficits*—All objective and subjective measures show unprecedented and unpredicted degradation relative to 1D Upright; 2) Marginal v*isual mitigation* – Redundant, veridical visual information marginally decreases blindfolded 2D self-balance deficits, whereas vision almost fully rescues 1D Supine Roll, a 0-g analog, balancing down to 1D Upright baseline; 3) *Upright 2D balancing with full gravitational cueing is worse than Supine 1D Roll without gravitational cueing*—Deficits in crash frequency, destabilizing commands, attentional demand, and mitigation by vision are more extreme during 2D Upright Roll & Pitch EC than during 1D Supine Roll EC; 4) *Learning* –2D balancing deficits can be fully or largely eliminated with practice, for all performance measures. The collective results are both surprising and uniquely relevant to adverse events, like aircraft crashes and falls from bipedal stance, and they justify the development of a new balancing paradigm and model.

## Data Availability

All data and analysis software are available by request from the corresponding author.
